# Understanding the molecular mechanisms of *Acori Tatarinowii* Rhizoma: *Nardostahyos* Radix et Rhizoma in epilepsy treatment using network pharmacology and molecular docking

**DOI:** 10.1097/MD.0000000000037224

**Published:** 2024-02-09

**Authors:** Guangyu Cheng, Xuan Wang, Chaojie Wang, Qi Zhang, Yiwen Zhang

**Affiliations:** a The First Affiliated Hospital Heilongjiang University of Chinese Medicine, Harbin, China; b Heilongjiang University of Chinese Medicine, Harbin, China.

**Keywords:** *Acori Tatarinowii* Rhizoma, epilepsy, molecular docking, *Nardostahyos* Radix et Rhizoma, network pharmacology

## Abstract

*Acori Tatarinowii* Rhizoma (ATR) and *Nardostahyos* Radix et Rhizoma (NRR) are well-known traditional Chinese medicines that have been extensively used for the treatment of epilepsy (EP). However, the precise molecular mechanism of ATR-NRR action remains unclear because of their intricate ingredients. This study aimed to investigate the underlying mechanism of ATR-NRR in EP treatment using network pharmacology and molecular docking techniques. Herbal medicine and disease gene databases were searched to determine active constituents and shared targets of ATR-NRR and EP. A protein-protein interaction network was constructed using the STRING database, while the Gene Ontology and the Kyoto Encyclopedia of Genes and Genome pathway enrichment were performed using R programming. An ingredient-target-pathway network map was constructed using the Cytoscape software, incorporating network topology calculations to predict active ingredients and hub targets. The binding abilities of active ingredients and hub targets were examined using molecular docking. Nine qualified compounds and 53 common targets were obtained. The prominent active compounds were kaempferol, acacetin, cryptotanshinone, 8-isopentenyl-kaempferol, naringenin, and eudesmin, while the primary targets were RELA, AKT1, CASP3, MAPK8, JUN, TNF, and TP53. Molecular docking analysis revealed that they have substantial binding abilities. These 53 targets were found to influence EP by manipulating PI3K-Akt, IL-17, TNF, and apoptosis signaling pathways. The findings of this study indicate that ATR-NRR functions against EP by acting upon multiple pathways and targets, offering a basis for future study.

## 1. Introduction

Epilepsy (EP), a widespread chronic neurological disorder that affects more than 65 million individuals of various ages worldwide, is complicated by a strong genetic predisposition and multiple risk factors (for example, fractures, intracranial infection, stroke, burns, and concussion).^[1]^ Approximately 90% of people suffering from EP are from developing regions.^[2]^ The incidence and prevalence of EP have shown an upward trend from 1990 to 2019, and its burden among the elderly population has also gradually increased, particularly in the western provinces of China.^[3]^ EP and its comorbidities, specifically depression, affect patients’ personal and physical health and quality of life, as well as cause heavy financial burdens to families and society.^[4,5]^

Antiseizure medication such as phenytoin, valproic acid, carbamazepine, and phenobarbital, the current primary treatment strategy, may suppress seizure occurrence in roughly two-thirds of individuals; however, it leads to certain side effects, including osteoporosis, sexual dysfunction, and vascular disease, and does not alter long-term prognoses.^[6]^

**Figure 1.**
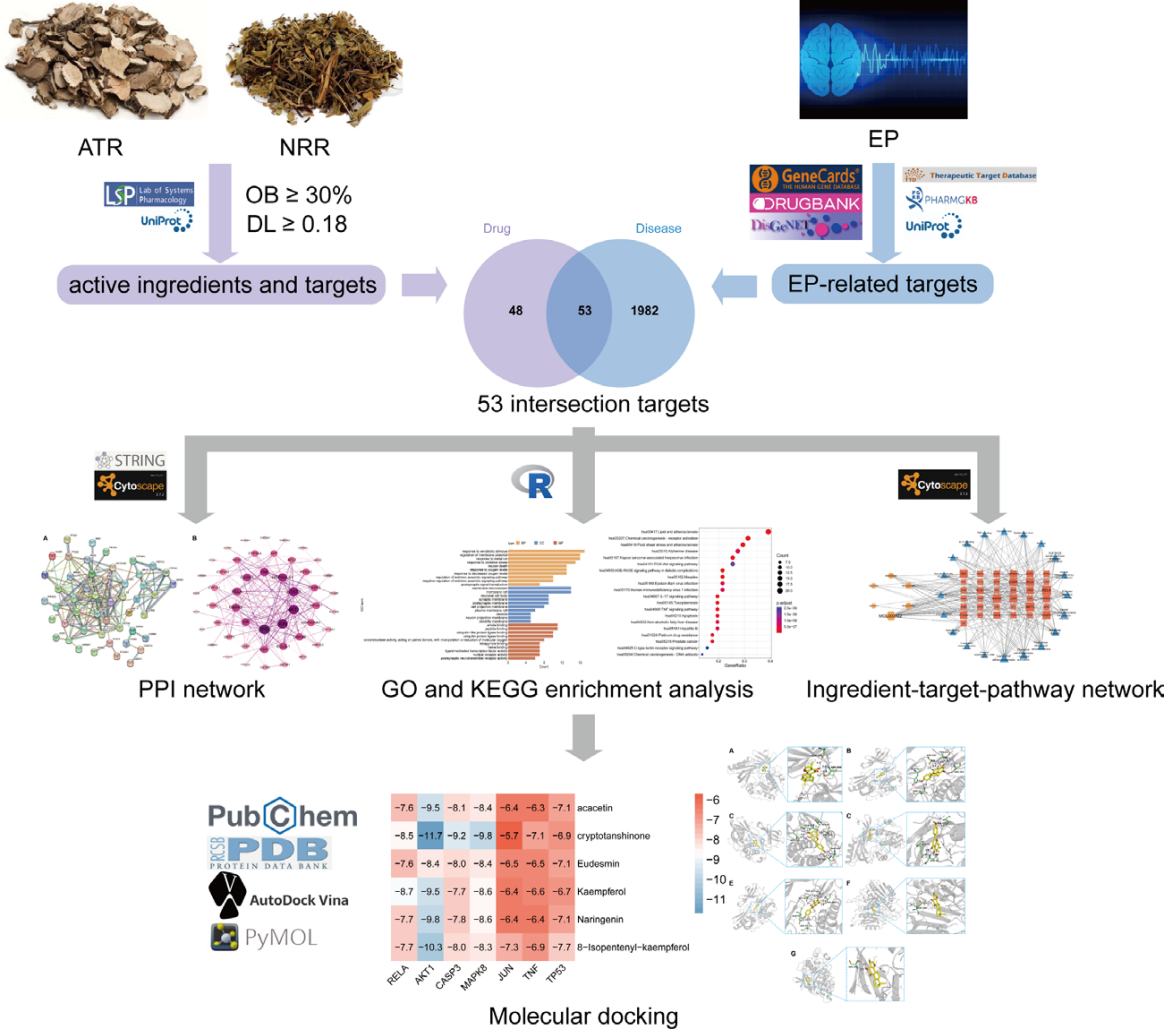
Network pharmacological and molecular docking study of ATR-NRR for the treatment of EP schematic diagram. ATR = *Acori Tatarinowii* Rhizoma, EP = epilepsy, GO = gene ontology, KEGG = Kyoto Encyclopedia of Genes and Genomes., NRR = *Nardostahyos* Radix et Rhizoma, PPI = protein-protein interaction.

Traditional Chinese Medicine (TCM) is a common alternative or supplementary therapy for EP. Studies have demonstrated its relative effectiveness and safety as a clinical strategy; therefore, the development of new TCM preparations for EP would be extremely valuable.^[7]^ *Acori Tatarinowii* Rhizoma (ATR; Shi Chang Pu in Chinese) is a useful TCM that was first documented in the Shennong Materia Medica. It belongs to the Araceae family and has shown efficacy in managing EP, depression, Alzheimer’s disease, and multiple mental health conditions.^[8–10]^ *Nardostahyos* Radix et Rhizoma (NRR; Gan Song in Chinese) is a perennial herbaceous medicinal plant belonging to the Valerianaceae family that can be used as a sedative, brain tonic, or mental rejuvenator and in the treatment of various neurological conditions such as EP and insomnia.^[11]^ However, most studies on these plant species have analyzed only a single target or pathway of ATR and NRR. The present investigation integrates network pharmacology and bioinformatics to reveal the molecular mechanism, necessary functional compounds, and associated physiological pathways of ATR-NRR in the treatment of EP as a whole (Fig. 1) and aims to provide a theoretical foundation and scientific basis for in-depth research on the pharmacological and material basis of ATR-NRR as well as related clinical and animal studies.

## 2. Materials and methods

### 2.1. Screening active components of ATR-NRR

The chemical composition of ATR and NRR was retrieved from the Traditional Chinese Medicine Systems Pharmacology (TCMSP, http://tcsmpw.com/tcsmp.php) database.^[12]^ The candidate compounds were screened using the thresholds of oral bioavailability (OB) ≥ 30% and drug similarity (DL) ≥ .18, and target prediction was performed for the selected compounds. The targets were input into Uniprot (http://www.uniprot.org/), specifying “*Homo sapiens*” as the biological species for normalization, and gene symbols and gene IDs for the target proteins were obtained.^[13]^

### 2.2. Acquisition of EP-related genes

The GeneCards (http://www.genecards.org/; relevance score > 2 × median), DrugBank (https://go.drugbank.com), Therapeutic Target Database (TTD) (http://db.idrblab.net/ttd/), pharmGKB (https://www.pharmgkb.org/), and DisGeNET (https://www.disgenet.org/) databases were searched using the keyword “epilepsy” to identify any possible targets associated with EP.^[14–18]^ Among them, the DrugBank and pharmGKB databases are constructed upon drug evidence derived from clinical trials, thereby offering a wealth of drug-related information and comprehensive details regarding drug targets. GeneCards, TTD, and DisGeNet databases draw upon literature as their foundation, with each database possessing distinctive strengths that mutually complement each other. EP-related targets identified across various databases were consolidated and dereplicated. Ultimately, the targets were standardized as gene names on UniProt.

### 2.3. Venn analysis of intersecting genes

The targets pertaining to potential ingredients and pathogenic targets associated with EP were integrated and plotted using the Evenn (http://www.ehbio.com/test/venn/#/) platform.^[19]^ The coinciding targets were exhibited within the overlapping domain.

### 2.4. Construction of the protein-protein interaction (PPI) network

To explore the direct (physical) and indirect (functional) relationships that compounds share with disease-related protein molecules from diverse perspectives, including biochemistry, genetic network, substance metabolism, and signal transduction, a PPI network was obtained for the intersection targets using the STRING11.5 (https://string-db.org) database, with the minimum interaction score set to a high confidence level (.7).^[20]^ The acquired PPI network association was visualized through CytoScape 3.7.2 software, utilizing the NetworkAnalyzer built-in tool to implement topology calculation and optimize the display of the PPI network diagram.

### 2.5. Gene ontology (GO) and kyoto encyclopedia of genes and genomes (KEGG) pathway enrichment

To investigate the roles of the overlapping target genes and their signaling pathways, “clusterProfiler,” “org.Hs.e.g..db,” and “ggplot2” R language packages were used for KEGG pathway and GO enrichment, encompassing the exploration of biological processes (BP), cellular components, and molecular functions, with *P* values set at < .01. Bubble and bar charts were used to showcase the top 20 KEGG pathways and the top 10 GO functions, respectively.

### 2.6. Construction of the active ingredient-target-pathway network

The top 20 pathways in the KEGG enrichment analysis and their pertinent component and target information were collated. We created the component-target-pathway network using Cytoscape 3.7.2 software and evaluated its topological parameters.

### 2.7. Molecular docking

Molecular docking simulations were performed to evaluate the intensity and mode of the interactions between the active ingredients and hub targets. After downloading the crystal structures of the pivotal targets’ protein receptors from the Protein Data Bank (http://www.rcsb.org/) database, these were imported into PyMol 2.2.0 software to remove the original ligands.^[21]^ The active ingredient structures as ligands were acquired from the PubChem compound database (http://pubchem.ncbi.nlm.nih.gov/). After eliminating the water molecules from the ligands and proteins, adding non-polar hydrogen bonds, and setting atomic type using the AutoDock software, the molecular ligands and proteins were converted into the “pdbqt” format. Molecular docking was performed using AutoDock Vina, and the semi-flexible docking method was employed, in which all docking parameters of the algorithm are set by default. The ionization energy was computed, and the minimum energy value was chosen as the docking affinity. Ultimately, the docked structures were visualized using the PyMol software.

## 3. Results

### 3.1. The active ingredients and potential targets of ATR-NRR

Nine active ingredients meeting the criteria of OB ≥ 30% and DL ≥ .18 were obtained from the TCMSP database (Table 1); 4 of them were active components for the ATR, and the remaining 5 pertained to NRR (Table 1).

**Table 1:** Main active ingredients of *Acori Tatarinowii* Rhizoma-*Nardostahyos* Radix et Rhizoma.

MOL ID	Molecular name	OB (%)	DL	Affiliated herbs
MOL003542	8-Isopentenyl-kaempferol	38.04	0.39	ATR
MOL003576	Eudesmin	52.35	0.62	ATR
MOL003578	Cycloartenol	38.69	0.78	ATR
MOL000422	Kaempferol	41.88	0.24	ATR
MOL001689	Acacetin	34.97	0.24	NRR
MOL010543	Acaciin	39.84	0.71	NRR
MOL000359	Sitosterol	36.91	0.75	NRR
MOL001040	Naringenin	42.36	0.21	NRR
MOL007088	Cryptotanshinone	52.34	0.4	NRR

ATR = *Acori Tatarinowii* Rhizoma, NRR = *Nardostahyos* Radix et Rhizoma.

### 3.2. The intersection genes of ATR-NRR and EP

**Figure 2.**
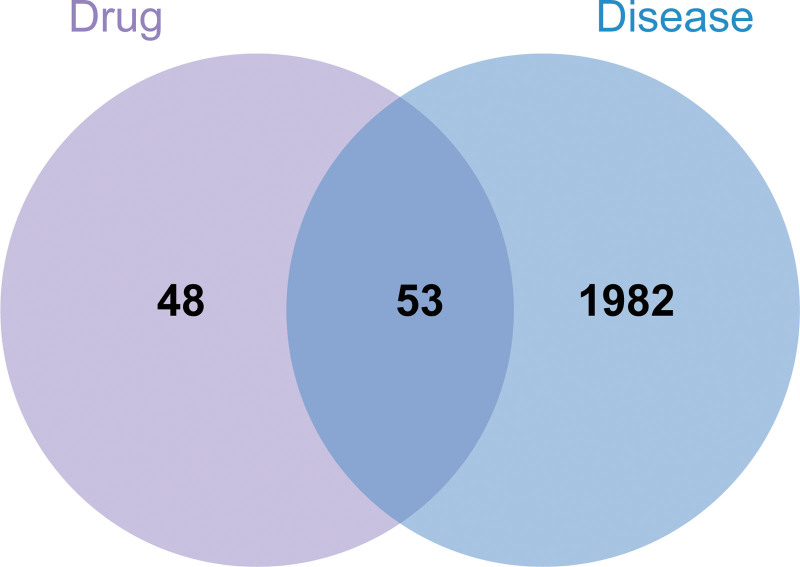
Venn diagram of *Acori Tatarinowii* Rhizoma (ATR)-*Nardostahyos* Radix et Rhizoma (NRR) and epilepsy (EP) gene targets. Purple and blue indicate ATR-NRR and EP, respectively.

Following investigation of the corresponding targets in the TCMSP database in accordance with the active ingredients, 53 annotated genes were collected through normalization of the target genes to the UniProt database while simultaneously filtering out the duplicate data. For EP, 2035 targets were retrieved from the GeneCards (relevance score > 1.64), DrugBank, TTD, pharmGKB, and DisGeNET databases. An intersection between ATR-NRR active ingredient targets and EP disease-related targets resulted in the identification of 53 potential target genes for ATR-NRR activity in EP (Fig. 2).

### 3.3. PPI

**Figure 3.**
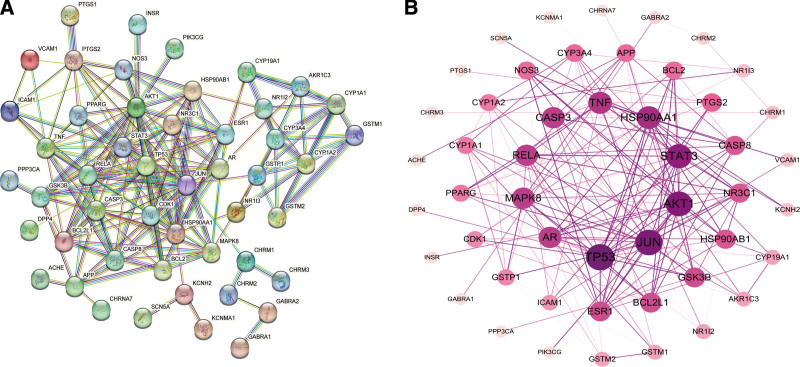
Protein-protein interaction (PPI) network of the *Acori Tatarinowii* Rhizoma (ATR)-*Nardostahyos* Radix et Rhizoma (NRR) against epilepsy (EP). (A) PPI networks exported from the STRING database. (B) PPI networks processed using the Cytoscape software. The top 10 targets in terms of degree value are situated at the epicenter of the PPI network, including TP53, JUN, AKT1, STAT3, HSP90AA1, TNF, CASP3, RELA, MAPK8, and AR.

A PPI network with 49 nodes (target proteins) and 188 edges (interactions) was initially constructed using the STRING database (Fig. 3A). The degree value serves as a metric for determining the centrality and significance of nodes in the PPI network. As a result, the PPI network was reconfigured using Cytoscape software, precisely sorting its nodes clockwise from highest to lowest, from deep to shallow, and from the center throughout, with reference to the degree value metric. Interaction strengths were represented by the vividness and thickness of edges (Fig. 3B).

### 3.4. Enriched GO and KEGG pathways

**Figure 4.**
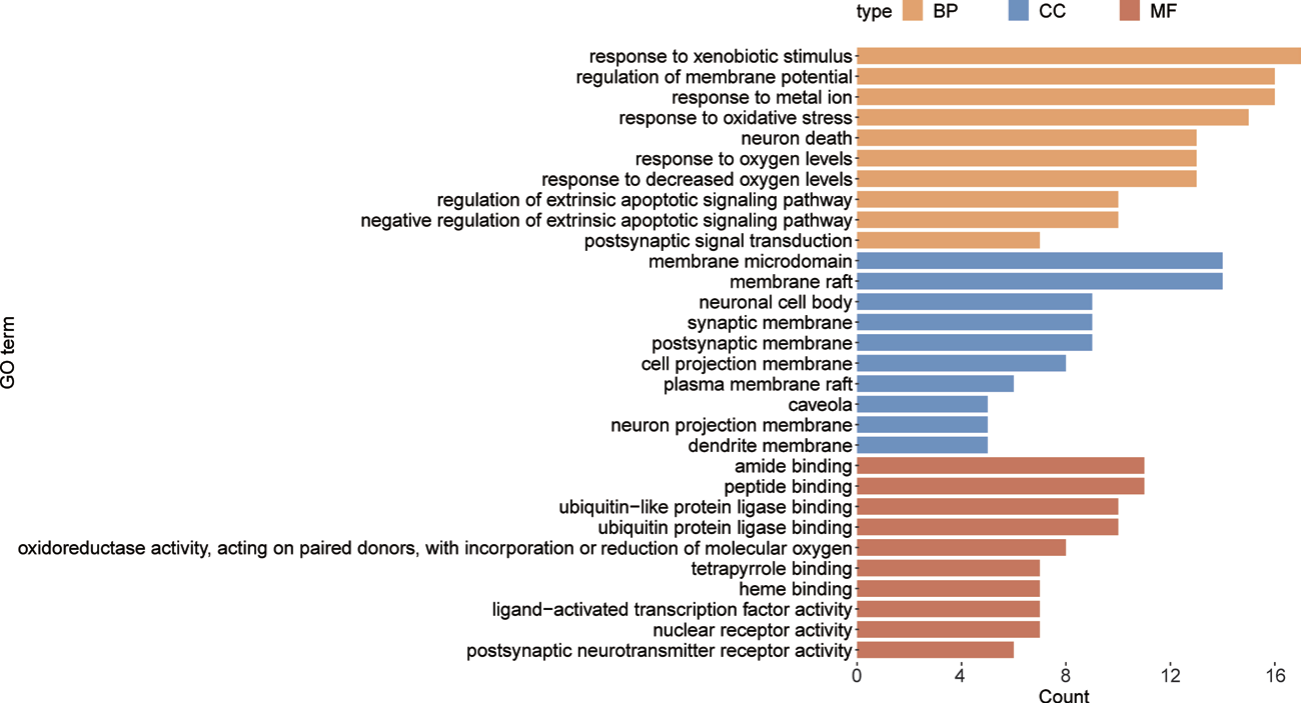
Top 10 significant GO enrichment entries of ATR-NRR in treating EP. BP = biological process, CC = cellular component, EP = epilepsy, GO = gene ontology, MF = molecular function.

**Figure 5.**
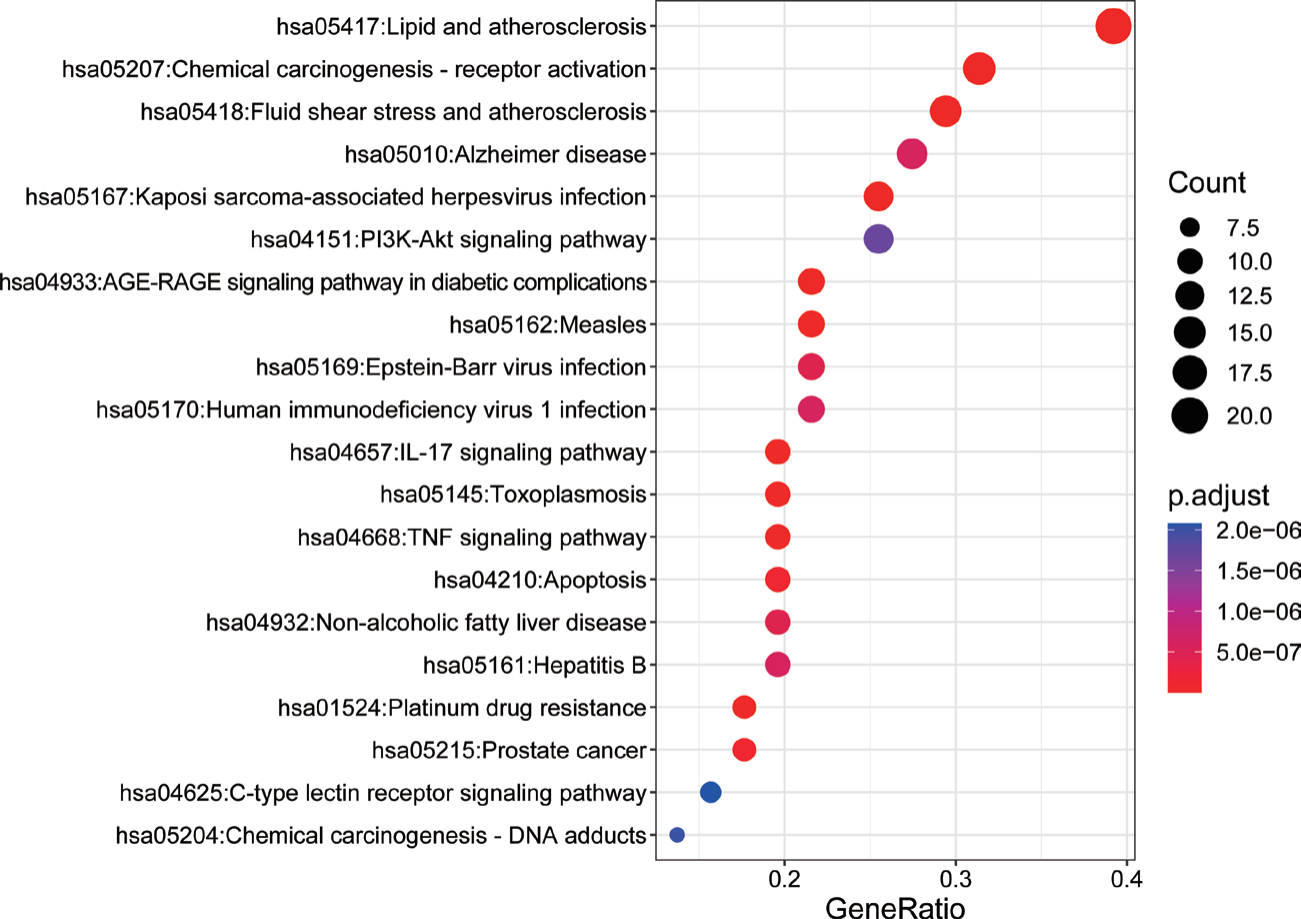
Top 20 significant KEGG enrichment entries of ATR-NRR in treating EP. EP = epilepsy, KEGG = Kyoto Encyclopedia of Genes and Genome pathway enrichment.

R language was utilized to perform GO and KEGG enrichment analysis for the intersection of ATR-NRR and EP, and the *P* value was set at .01. BP was primarily associated with the regulation of membrane potential, response to oxidative stress, neuronal death, response to oxygen levels, regulation of the extrinsic apoptotic signaling pathway, and postsynaptic signal transduction (Fig. 4). The neuronal cell body and synaptic membrane were enriched as cellular components. Molecular function was mainly related to ligand-activated transcription factor and postsynaptic neurotransmitter receptor activities. Additionally, the top 20 KEGG pathways based on *P* values included predominantly Lipid and atherosclerosis, Chemical carcinogenesis - receptor activation, Fluid shear stress and atherosclerosis, Alzheimer’s disease, Kaposi sarcoma-associated herpesvirus infection, the phosphatidylinositol 3 kinase (PI3K)-Akt, AGE-RAGE signaling pathway in diabetic complications, Measles, Epstein-Barr virus infection, Human immunodeficiency virus 1 infection, interleukin (IL)-17, and tumor necrosis factor (TNF) signaling pathways and apoptosis (Fig. 5).

### 3.5. Active ingredient-target-pathway network diagram

**Figure 6.**
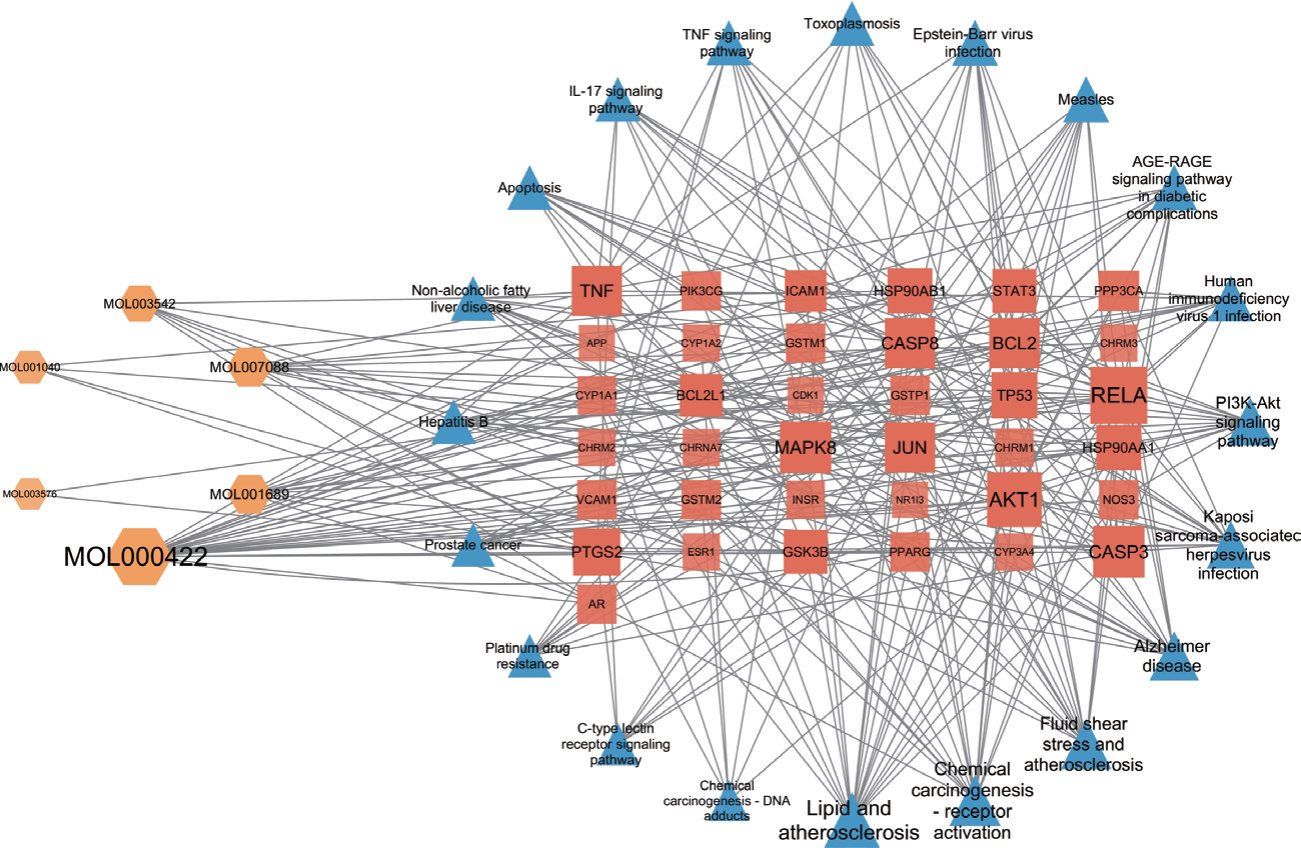
Ingredient-target-pathway network diagram of *Acori Tatarinowii* Rhizoma (ATR)-*Nardostahyos* Radix et Rhizoma (NRR) intervention in epilepsy (EP). The orange hexagon, red square, and blue triangle represent the active ingredients, target genes, and pathways, respectively. The larger the degree value, the darker the color and the larger the shape.

Analysis of the network topology parameters of the component-target-pathway network using the built-in tools of CytoScape revealed key components and hub genes. Each active component in this network analysis was connected to several targets, and each target corresponded to numerous components (Fig. 6), implying the presence of diverse ingredients within ATR-NRR that might potentially ameliorate EP through multiple targets. The principal therapeutic compounds included kaempferol, acacetin, cryptotanshinone, 8-isopentenyl-kaempferol, naringenin, and eudesmin. RELA, AKT1, CASP3, MAPK8, JUN, TNF, and TP53 exhibited superior status in terms of degree values in this network in addition to being the top 10 targets in the PPI network, indicating that they are core genes for ATR-NRR intervention in EP.

### 3.6. Molecular docking results

**Figure 7.**
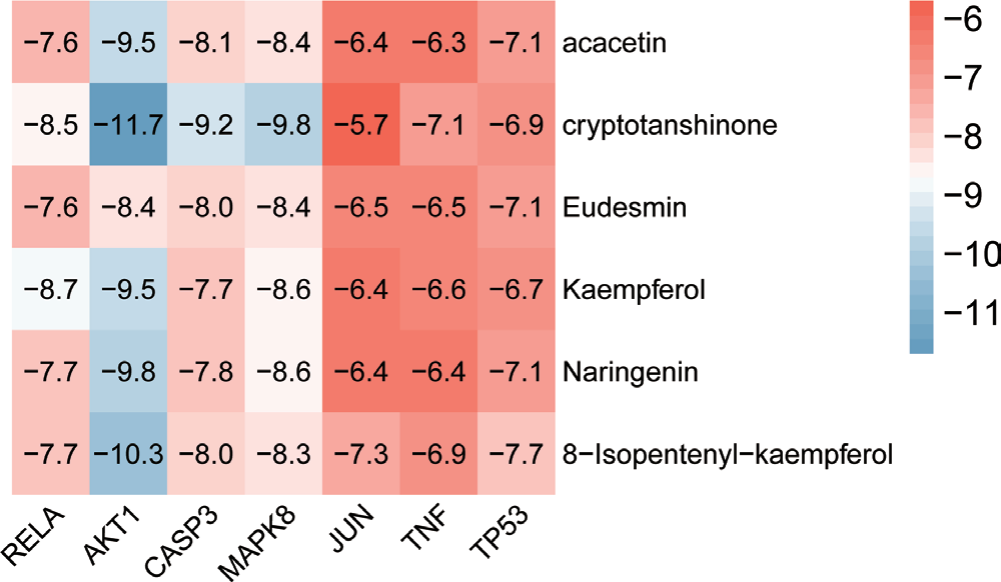
Molecular docking heatmap.

**Figure 8.**
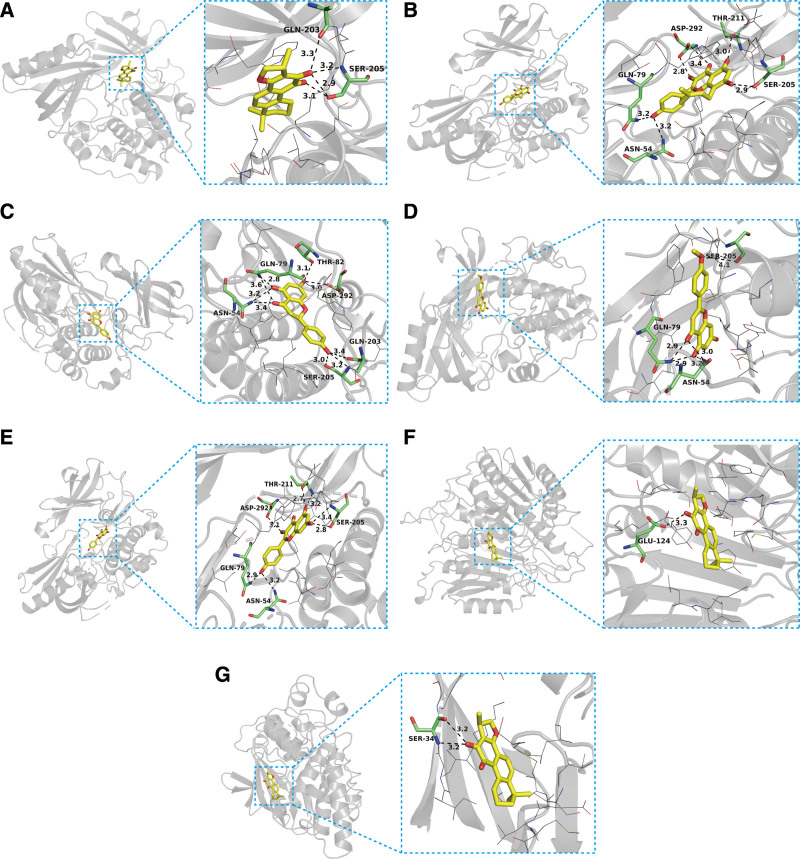
Molecular docking diagram of the interactions with strong binding activity (docking score < −9 kcal/mol). The docking of (A) cryptotanshinone with AKT1, (B) 8-isopentenyl-kaempferol with AKT1, (C) Naringenin with AKT1, (D) acacetin with AKT1, (E) Kaempferol with AKT1, (F) cryptotanshinone with CASP3, and (G) cryptotanshinone with MAPK8.

Based on the network pharmacology findings, we investigated 6 crucial compounds for molecular docking with 9 core targets to examine their interactions at a deeper level. We categorized the binding activity as standard and strong when the binding affinity was <−4.25 and <−7.0 kcal/mol, respectively.^[22]^ The average docking affinity was found to be − 7.79 kcal/mol, with 71.42% of the total results attaining a docking affinity of < −7.0 kcal/mol (Fig. 7). Cryptotanshinone was found to exhibit the highest intermolecular stability with target AKT1 (docking score = −11.7 kcal/mol). Based on the scoring outcomes, ATR-NRR active ingredients displayed a good binding capability towards the core targets. The outcomes stemming from selected binding energies that were < −9 kcal/mol were visualized using PyMol (Fig. 8).

## 4. Discussion

Episodic manifestation of transient psychiatric, sensory, motor, and autonomic indications that result from anomalous and excessive or synchronous firing of neurons within the brain is the defining characterization of an epileptic seizure.^[23]^ Recurrent seizures may cause nearly half of patients to experience neurologic, cognitive, somatic, or medical disorders, such as anxiety, attention deficit disorders, type 1 diabetes, and migraines.^[24]^ Several theories have been proposed regarding mechanisms of EP occurrence and progression, including acute changes such as transcriptional events, oxidative stress, neuronal death, the initiation of inflammatory cascades, and chronic anatomical alterations such as neurogenesis, synaptic plasticity, and gliosis.^[25]^ This indicates the intricacies underlying the pathogenesis of EP, with manifold targets and interrelated pathways, precluding the ability of a singly targeted pharmacological remedy to attain the desired therapeutic outcome. Chinese medicines possess the potential to manifest synergistic effects due to their multiple pharmacologically active compounds, which pervade several targets and pathways. Additionally, their distinguished safety profile and minimal side effects confer significant advantages for the treatment of conditions like EP that mandate prolonged therapeutic intervention. One of our previous studies has revealed that ATR-NRR possesses the potential to ameliorate EP; however, the antiepileptic mechanism and primary active constituents have not been fully elucidated. In the present study, we explored the mechanism of underlying ATR-NRR in the treatment of EP through an analysis of network pharmacology and molecular docking.

The analysis of active “ingredient-target-pathway” networks demonstrates that ATR-NRR constituents, including kaempferol, acacetin, cryptotanshinone, 8-isopentenyl-kaempferol, naringenin, and eudesmin, exert their effects on multiple targets and pathways associated with EP, indicating that these active components may play a pivotal role in therapy and need further investigation. Kaempferol has demonstrated a versatile ability to provide protection to the nervous system by modulating several proinflammatory signaling pathways, including p38 mitogen-activated protein kinases, serine/threonine kinase (AKT), and β-catenin cascades.^[26]^ The overabundance of glutamate release appears as a crucial element in the genesis of neuronal damage in EP, and acacetin exhibits a selective ability to hinder glutamate release from rat hippocampal nerve terminals. Furthermore, acacetin mitigates neural cell demise and microglial activation in the CA3 zone of the hippocampus, ensuing from kainic acid-induced neural degeneration.^[27]^ Cryptotanshinone could exert its neuroprotective capabilities by demonstrating antioxidant properties.^[28]^ 8-prenylkaempferol exhibits efficacy in reducing pro-inflammatory nitric oxide induction as it significantly impedes lipopolysaccharide-induced nitric oxide production and c-Jun phosphorylation in macrophages.^[29]^ Naringenin treatment reinstates hippocampal antioxidant status and mitigates lipid peroxidation in epileptic mice, decreasing seizure severity.^[30]^ Eudesmin exhibits notable sedative and anticonvulsant potentials, and the modus operandi of eudesmin could be associated with decreased CASP3 and amplified Bcl-2 expression, resulting in anti-apoptotic effects in neurons within the brain.^[31]^

Based on the PPI network analysis and topology algorithms implemented in Cytoscape software, in conjunction with the “component-target-pathway” network analysis, it was determined that RELA, AKT1, CASP3, MAPK8, JUN, TNF, and TP53 have been identified as the hub target proteins in the treatment of EP. As a member of the NF-κB transcription factor family, RELA has regulatory functions in immune responses, inflammatory processes, cellular proliferation, sustenance, and apoptosis and constitutes a pivotal determinant of epileptic pathophysiology, leading to the hypothesis that modulating NF-κB signaling could potentially serve as a secure and effective approach for treating for EP.^[32]^ AKT1 is a serine/threonine kinase with paramount roles in many physiological and pathological processes, most notably in epileptogenesis.^[33]^ CASP3 plays a key role in apoptosis following epileptic seizures and is integrally associated with neurodegeneration, neurogenesis, synaptogenesis, and astrocyte formation during the nascent phases of EP.^[34]^ The absence of MAPK8 (JNK1) has been demonstrated to mitigate the onset and gravity of seizures while concurrently decreasing glial responsiveness, constraining pro-inflammatory gene expression, and impeding neuronal damage.^[35]^ The anomalous manifestation of JUN exacerbates inflammatory processes, harming the nervous system and inducing various neurological symptoms. The augmented expression of JUN is also related to the onset of EP.^[36]^ TNF is an inflammatory cytokine; a clinical investigation has reported that its concentrations are elevated in individuals with severe EP compared to those with milder forms of EP and non-afflicted individuals, revealing a plausible correlation between the levels of TNF-α and the severity of EP.^[37]^ As a multifaceted protein, TP53 regulates cellular processes such as the cell cycle, DNA repair, and apoptosis. Studies on seizures during EP have shown an up-regulation of TP53 and CASP3 in the brain; genetic elimination or pharmacologic suppression of TP53 can alleviate seizure-induced cell apoptosis.^[38]^

BPs enriched through GO analysis include responses to oxidative stress, neuronal death, regulation of the extrinsic apoptotic signaling pathway, and postsynaptic signal transduction. The KEGG enrichment analysis indicates that the signaling pathways of ATR-NRR intervention in EP are mainly related to oxidative stress, inflammation, programmed cell death pathways, including the PI3K-Akt, IL-17, TNF, and apoptosis signaling pathways. The PI3K/AKT signaling cascade orchestrates signal transduction and multiple BPs, including cell propagation, apoptosis, and metabolism, and has been demonstrated to contribute crucially to the central nervous system.^[39]^ Studies report that oxidative stress stemming from EP might be relieved by activating the PI3K/Akt pathway, where apoptosis and detrimental effects are inhibited.^[40]^ Notably, the effects of IL-17 on neuroinflammation, blood-brain barrier integrity, and the secretion of neurotransmitters have been identified as crucial factors in the onset and evolution of EP.^[41]^ The activation of the IL-17 pathway has been indicated to contribute to the epileptogenicity of cortical abnormalities in focal cortical dysplasia, implying that it may be a novel potential target for antiepileptic therapies.^[42]^ The TNF pathway initiation escalates the concentration of the pro-inflammatory mediator RELA (p65), which promotes neuroinflammation and has a branch involved in regulating apoptosis or necroptosis.^[43]^ Apoptosis signaling pathways are significantly linked to neuronal death following seizures and epileptogenesis, making them a crucial area of evolving research aimed at mitigating the detrimental effects of seizures on the brain.^[44]^

This study has some limitations. First, our research is based on numerous online databases; consequently, the depth of basic research and the precision and promptness of the data in each database can produce bias. Second, the examination of the active compounds of ATR-NRR did not include the analysis of their content, absorption pathway, or metabolic form. Finally, we employed solely up-to-date bioinformatics methods to initiate an exposition of the mechanism of ATR-NRR in treating EP using network pharmacology and molecular docking. Therefore, future research should ascertain, through in vivo and in vitro experimentation, aided by contemporary analytical instruments and pharmacokinetic methods, the exact efficacious constituents, primary regulatory targets, and pathways of ATR-NRR employed in the management of EP. Nevertheless, the outcomes of this study offer commendable directions for the design of our experimental undertaking.

## 5. Conclusion

This study revealed that active compounds in ATR-NRR, such as kaempferol, acacetin, cryptotanshinone, 8-isopentenyl-kaempferol, naringenin, and eudesmin, may act on potential hub targets, including RELA, AKT1, CASP3, MAPK8, JUN, TNF, and TP53, regulating several pathways, such as PI3K-Akt, IL-17, TNF, and apoptosis signaling pathways. The molecular docking analysis findings offer theoretical evidence indicating that the identified proteins could likely serve as key targets for therapeutics in managing EP. This confirmed the hypothesis that ATR-NRR exhibits a multi-component, multi-target, and multi-pathway integrated effect, suggesting novel ways of studying the potential mechanism underlying ATR-NRR’s therapeutic effects on EP. These findings furnish a point of reference for the clinical management of EP and establish a theoretical foundation for the advancement of novel antiepileptic medications encompassing diverse pathways.

## Author contributions

**Data curation**: Guangyu Cheng.

**Formal analysis**: Guangyu Cheng, Yiwen Zhang.

**Methodology:** Guangyu Cheng, Yiwen Zhang, Xuan Wang, Qi Zhang, Chaojie Wang.

**Investigation**: Xuan Wang, Qi Zhang, Chaojie Wang.

**Funding acquisition**: Guangyu Cheng.

**Writing – original draft**: Guangyu Cheng, Yiwen Zhang.

**Writing – review & editing**: Guangyu Cheng, Yiwen Zhang.
